# Influencing factors of peripheral blood indicators on 30-day mortality risk in children with hemophagocytic lymphohistiocytosis based on weighted quantile sum regression: a single-center retrospective cohort study

**DOI:** 10.3389/fped.2026.1862505

**Published:** 2026-07-15

**Authors:** Chuncan Wu, Xiaoying Zhang, Xiang Lan, Weijun Huang, Zhonglv Ye, Lili Liu, Chuan Tian

**Affiliations:** Department of Pediatrics, Affiliated Hospital of Guangdong Medical University, Zhanjiang, China

**Keywords:** 30-day mortality risk, hemophagocytic lymphohistiocytosis, pediatric, peripheral blood parameters, weighted quantile sum regression

## Abstract

**Objective:**

To investigate the joint effect of peripheral blood parameters on the 30-day mortality risk in children with hemophagocytic lymphohistiocytosis (HLH) using weighted quantile sum (WQS) regression, and to elucidate the clinical weights of each feature.

**Methods:**

A retrospective cohort design was adopted. A total of 133 pediatric HLH patients diagnosed at the Children's Medical Center of the Affiliated Hospital of Guangdong Medical University between January 1, 2015, and December 30, 2024, were included. The patients were divided into a non-survival group (*n* = 29) and a survival group (*n* = 104) based on their 30-day survival outcomes following diagnosis. Baseline peripheral blood parameters within 24 h prior to or on the day of diagnosis were collected. After preliminary screening via univariate logistic regression, a WQS regression model was utilized to evaluate the joint effect of peripheral blood parameters and extract empirical weights. In addition, restricted cubic spline (RCS) analysis was employed to explore the dose-response relationship between core parameters and mortality risk. The predictive efficacy and clinical net benefit of the core parameters and their combined model were evaluated using receiver operating characteristic (ROC) curves and decision curve analysis (DCA).

**Results:**

Univariate analysis indicated that red blood cell distribution width (RDW), mean corpuscular hemoglobin (MCH), hematocrit (HCT), and hemoglobin (Hb) were significantly associated with early mortality risk. The negative-direction WQS regression model revealed that the overall improvement of peripheral blood parameters had a significant joint protective effect against 30-day mortality risk (*β* = −0.936, *P* = 0.012), with MCH (weight 62.6%) and HCT (weight 29.8%) exhibiting the highest relative weights within the evaluated mixture. RCS analysis demonstrated a significant overall association between MCH, HCT, and mortality risk, approximating a linear relationship. The area under the curve (AUC) of the combined predictive model constructed by MCH and HCT improved to 0.728, indicating superior predictive efficacy over single indicators. Moreover, DCA confirmed that the combined predictive model provided a favorable clinical net benefit across a broad range of threshold probabilities compared to single parameters.

**Conclusion:**

The analytical strategy incorporating the WQS regression model effectively mitigates collinearity interference among clinical variables. Abnormalities in peripheral blood parameters, primarily driven by HCT and MCH, serve as a exploratory composite indicator for predicting early fatal outcomes in pediatric HLH, offering a potential early warning signal for critical triage in the early stages of the disease.

## 1 Introduction

Hemophagocytic lymphohistiocytosis (HLH) in children is an immune dysregulation syndrome triggered by predisposing factors such as genetic defects, infections, malignancies, or autoimmune diseases. Its core pathological mechanism lies in the functional defects of cytotoxic T lymphocytes and natural killer cells, leading to the overactivation of macrophages and the massive secretion of pro-inflammatory cytokines, which subsequently trigger a cytokine storm and multiple organ dysfunction syndrome (1, 2). With the popularization of the HLH-2004 immunochemotherapy regimen and the maturation of hematopoietic stem cell transplantation techniques, the 5-year overall survival rate of pediatric patients has reached approximately 62% (3). However, this improvement in long-term prognosis has not entirely eliminated the perilous characteristics of the acute phase of the disease. Studies have shown that approximately 20% to 25% of patients still die before hematopoietic stem cell transplantation or during the early stages following diagnosis (4). Within the first 30 days after diagnosis, patients often face an extremely high mortality risk due to severe coagulopathy, central nervous system involvement, or refractory infections (5). Clinical practice indicates that pediatric HLH exhibits a high degree of phenotypic heterogeneity, with significant differences among patients regarding onset acuity, the extent of organ involvement, and response to initial treatment. During the acute phase of critical illness, routine peripheral blood parameters serve as foundational indicators of the body's oxygen-carrying capacity and bone marrow suppression. They also act as crucial markers for severe systemic inflammatory cascades, microvascular thrombosis, and acute endothelial injury. Traditional prognostic evaluation systems mostly rely on linear thresholds of single biomarkers (e.g., ferritin, fibrinogen, soluble CD25) or traditional regression models (6). These methods assume a homogeneous linear effect of risk factors across the entire population, making it difficult to capture the complex fluctuations of systemic inflammatory networks and non-linear interactions among variables. Therefore, systematically analyzing routine peripheral blood features helps uncover their dynamic patterns and joint mechanisms. This approach offers significant clinical value for identifying specific early mortality risk trajectories in pediatric HLH. In the real-world pathophysiological environment of critical clinical conditions, peripheral blood parameters rarely change in isolation; instead, they exhibit significant multicollinearity. When employing traditional multivariable logistic regression or Cox proportional hazards models, intense collinearity interference among variables will significantly inflate the standard errors of parameter estimates, leading to distorted model predictive efficacy and potentially causing the erroneous exclusion of key variables with genuine core clinical prognostic value (7). To overcome these traditional statistical limitations, weighted quantile sum (WQS) regression offers an innovative modeling strategy. WQS can perform quantile step transformations on multiple continuous variables with collinear features and quantitatively evaluate the overall comprehensive exposure level by constructing a weighted index in a pre-specified single fixed direction (positive harmful or negative protective effect). This method not only fundamentally circumvents the statistical dimensional bias caused by multicollinearity but also estimates the relative statistical contribution of each clinical parameter within the joint exposure through internal empirical weights (8, 9). In a large-sample cohort study published in 2023, Requena successfully utilized the WQS model to evade collinearity interference among high-dimensional biochemical markers, elucidating the joint network effects of mixed environmental exposures on serum cytokines and acute-phase inflammatory response proteins (10). In the field of cardiovascular clinical research, Zhang (11) leveraged the WQS framework to assess the specific directional contributions and joint exposure weights of blood cell parameters in the risk of incident coronary heart disease. However, the application of WQS regression combined with blood cell parameters in the field of pediatric HLH remains scarce. Based on this, the present study utilized the WQS regression model to investigate the intrinsic joint effect characteristics of peripheral blood red blood cell parameter clusters at the time of HLH diagnosis in children, aiming to provide a more scientific hematological evidence base for early fatal warning in pediatric HLH and to offer decision-making support for early clinical intervention.

## 2 Materials and methods

### 2.1 Study population

This study employed a retrospective cohort design. Children diagnosed with HLH who were hospitalized at the Children's Medical Center of the Affiliated Hospital of Guangdong Medical University between January 1, 2015, and December 30, 2024, were selected as study subjects. For children with multiple hospitalizations, only data from the first admission were included.

Inclusion criteria: (1) Age<18 years; (2) Diagnostic criteria: strict adherence to the HLH-2004 diagnostic guidelines published by the Histiocyte Society (12), whereby patients must fulfill molecular biological diagnosis (presence of known HLH-related gene mutations) or meet at least 5 of the following 8 clinical and laboratory criteria: ① fever; ② splenomegaly; ③ cytopenias (affecting two or three lineages in peripheral blood); ④ hypertriglyceridemia and/or hypofibrinogenemia; ⑤ hemophagocytosis identified in bone marrow, spleen, or lymph nodes; ⑥ low or absent natural killer (NK) cell activity; ⑦ serum ferritin (SF) ≥500 mu g/L; ⑧ elevated soluble CD25 (sCD25) level (≥2,400∼U/mL).

Exclusion criteria: (1) Prior intervention interference: patients who had undergone hematopoietic stem cell transplantation (HSCT) treatment before enrollment, as their immune baseline has fundamentally altered; (2) Missing key outcomes: patients lost to follow-up after discharge or whose medical records could not trace survival outcomes within 30 days after diagnosis; (3) Severe data deficiency: core peripheral blood indicators (such as complete blood count, coagulation function, etc.) with missing rates exceeding 20% that cannot be effectively repaired through imputation techniques.

This study strictly adhered to the ethical principles of the Declaration of Helsinki and its revised versions. The research protocol was submitted to and approved by the Medical Ethics Committee of the Affiliated Hospital of Guangdong Medical University (Ethics Approval No.PJKT2026-029). Given that this study is a retrospective observational study that only collects and analyzes existing clinical medical records without intervening in patient treatment plans, and all personal privacy information of subjects has been strictly de-identified during the research process with controllable risks, the Ethics Committee approved a waiver of informed consent after deliberation.

### 2.2 Outcome definition and grouping

The primary endpoint of this study was defined as all-cause mortality within 30 days from the date of HLH diagnosis. Information on patient survival status was obtained through review of the hospital electronic medical record system combined with telephone follow-up, with the follow-up cutoff date being 30 days after diagnosis or the date of death occurrence. Based on 30-day survival outcomes, the study cohort was divided into a non-survivor group and a survivor group. To ensure accuracy of the prediction model and exclude interference of treatment interventions on biomarkers, all laboratory indicators included in the analysis (including complete blood count, coagulation function, and biochemical indicators) were strictly limited to baseline data collected on the day of diagnosis or within 24 h before diagnosis, and all samples were collected before patients received HLH-specific induction therapy (such as dexamethasone, etoposide, or cyclosporine).

### 2.3 Laboratory measurements

The collection time window for all laboratory parameters included in the analysis was strictly limited to the day of HLH diagnosis or within 24 h prior to diagnosis. To ensure the homogeneity and accuracy of the test data, blood sample collection was performed by uniformly trained professional nursing staff. If multiple test records existed within the specified time window, the value closest to the time of HLH diagnosis and prior to specific induction therapy was selected as the baseline data for analysis to objectively reflect the patient's initial immune-inflammatory status.

All specimens were processed strictly in accordance with clinical laboratory standard operating procedures. Complete blood count samples were analyzed using an automated hematology analyzer (Sysmex XN-3000 series). This study focused on extracting and recording the basic demographic characteristics (sex, age) of the patients, as well as complete blood count-related parameters, including white blood cell count (WBC), absolute neutrophil count (ANC), lymphocyte count (LYM), monocyte count (Monocytes), red blood cell count (RBC), hemoglobin (Hb), hematocrit (HCT), mean corpuscular hemoglobin (MCH), mean corpuscular hemoglobin concentration (MCHC), red blood cell distribution width (RDW), reticulocyte percentage (Reticulocyte), plateletcrit (PCT), and platelet count (PLT). To enable rigorous multivariable clinical adjustment in the subsequent prediction models, we also systematically extracted core clinical covariates reflecting disease severity and coagulopathy, specifically including intensive care unit (ICU) admission, central nervous system (CNS) involvement, prothrombin time (PT), and activated partial thromboplastin time (APTT).

### 2.4 Sample size calculation

**Figure 1:**
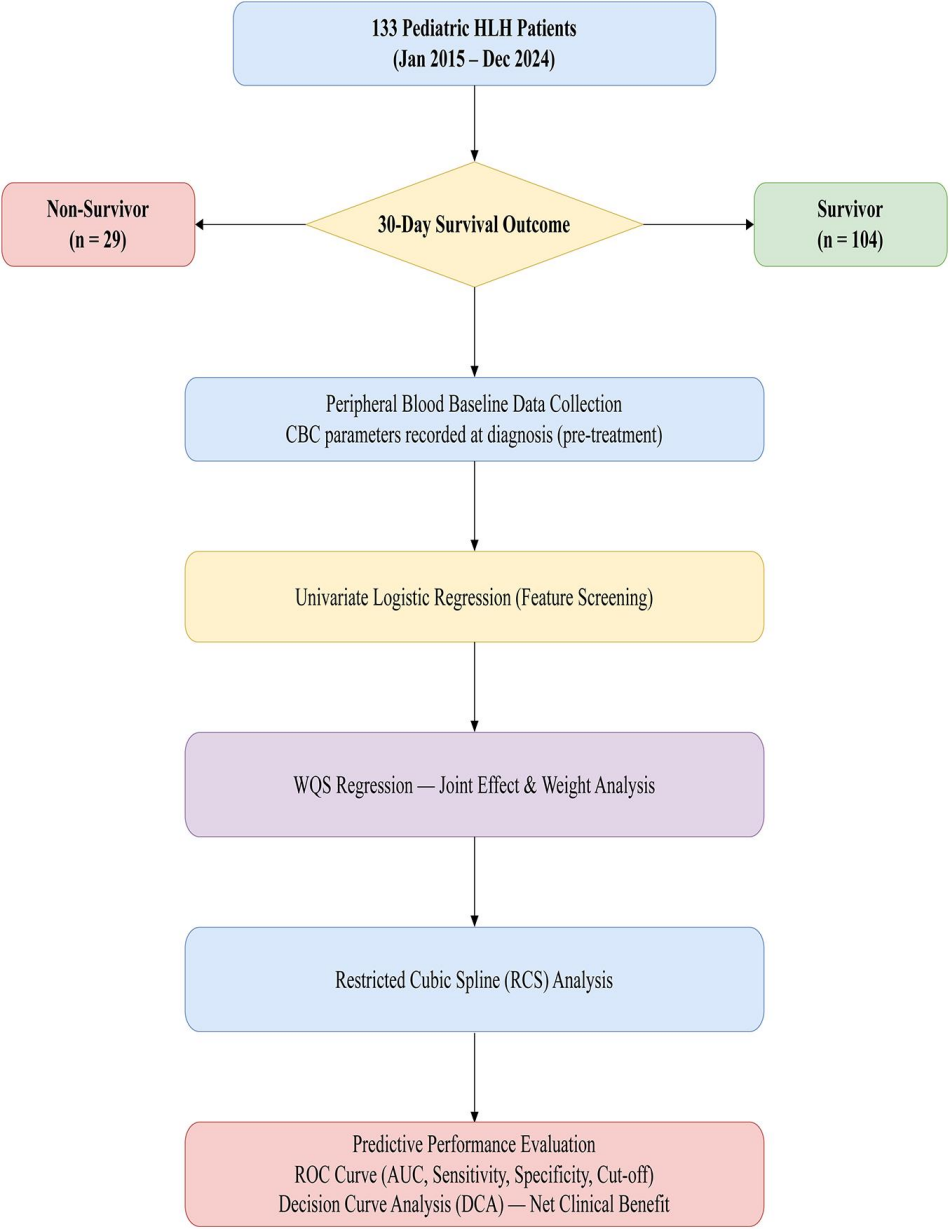
Flowchart of the overall study design and patient selection process. The workflow delineates the temporal sequence from the retrospective inclusion of 133 pediatric patients with HLH, through the collection of baseline peripheral blood parameters, to the sequential statistical analyses including univariate screening, WQS, RCS, and the ultimate clinical utility evaluations via ROC and DCA. HLH, hemophagocytic lymphohistiocytosis; WQS, weighted quantile sum; RCS, restricted cubic spline; ROC, receiver operating characteristic; DCA, decision curve analysis.

Sample size estimation in this study was based on the incidence estimation formula for cohort studies. Referring to relevant literature and clinical data, the short-term mortality rate of pediatric patients was assumed to be approximately 20% (*P* = 0.20). To ensure statistical power, confidence level was set at 95% $\left(\alpha =0.05,\;Z_{1-\alpha /2}=1.96\right)$. Considering that this study is a retrospective analysis, the allowable error was set at 7.5%. The pmsampsize package in R was used to assist calculation, with the sample size calculation formula as follows: $N=\frac{Z^{2}\times P(1-P)}{\delta^{2}}=\frac{1.96^{2}\times 0.20\times (1-0.20)}{0.075^{2}}\approx 109.$ Based on the above formula, the initial sample size was calculated to be 109 cases. Considering possible information loss or matching attrition during retrospective data collection, a 10% sample attrition rate was set. $N_{\;final}=\frac{N}{1-0.10}=\frac{109}{0.9}\approx 121.$ The final estimated required sample size was approximately 121 cases. T This study ultimately included 133 pediatric patients who met the inclusion criteria, exceeding the theoretically estimated sample size for mortality incidence observation (Figure 1). However, it is important to acknowledge that this initial calculation was not *a priori* powered for formal multivariable prediction modeling. Appropriate sample size estimation for prediction models should ideally consider the number of candidate predictors against expected outcome events to address model shrinkage, optimism, and calibration precision. Given the retrospective design, our sample size was inherently constrained by the available patient population during the study period. With 29 mortality events, the events-per-variable ratio is limited; therefore, the predictive modeling and related evaluations in this study are strictly exploratory.

### 2.5 Data preprocessing and missing data handling

Prior to formal statistical analysis, raw data were rigorously evaluated for missing values and outliers to ensure dataset integrity. The proportion of missing data for each variable was explicitly assessed. Demographic and disease characteristics (e.g., sex, age, and etiology) had completely intact data (0.00% missing). For routine complete blood count parameters (including WBC, ANC, RBC, Hb, HCT, MCH, MCHC, RDW, PCT, and PLT) and coagulation function covariates (PT, APTT), the missing data proportion was 5.56%. Specifically, missing values were imputed using the Multiple Imputation by Chained Equations (MICE) algorithm, incorporating all relevant baseline covariates and the outcome variable into the imputation model to ensure robust estimations. Outliers were defined using the interquartile range (IQR) method as values falling below Q1-1.5×IQR or above Q3 + 1.5×IQR, and only verified data entry errors or implausible laboratory artifacts were excluded.

### 2.6 Statistical analysis

All statistical analyses were performed using R software version 4.4.2. Prior to analysis, raw data were cleaned and subjected to quality control to ensure dataset integrity. For baseline characteristics, normally distributed continuous variables were expressed as mean ± standard deviation $\left(\bar{x}\pm s\right)$ and compared between groups using the independent samples *t*-test. Non-normally distributed continuous variables were presented as median and interquartile range [*M* (*P25*, *P75*)] and compared using the Mann–Whitney *U*-test. Categorical variables were expressed as frequencies and percentages [*n* (%)] and compared using the Chi-square test or Fisher's exact test. Initially, univariate logistic regression analysis was utilized to preliminarily screen for prognosis-related variables. Although WQS regression is theoretically capable of handling multicollinear mixtures without pre-screening, applying this initial filtering step was a strict statistical necessity rather than a mere preference. According to the established events-per-variable (EPV) criterion, our limited number of mortality events (*n* = 29) tightly restricts the total degrees of freedom available for multivariable modeling. Forced inclusion of all candidate peripheral blood parameters, alongside necessary clinical covariates required for multivariable adjustment, would severely violate the EPV threshold, leading to substantial over-parameterization. In the context of WQS modeling, such high dimensionality relative to the limited event count increases the risk of non-convergence for the non-linear optimization solver during the bootstrap step, potentially generating computational noise and rendering the empirical weights unstable. Therefore, this univariate screening acted as a practical dimension-reduction strategy. It allowed the WQS algorithm to prioritize delineating the joint effects among variables with potential marginal clinical relevance, thereby supporting algorithmic convergence and enhancing the reliability of the final component weights. Considering the high physiological correlation and multicollinearity among the included peripheral blood parameters, the WQS regression model was employed. To ensure strict methodological transparency and reproducibility, the detailed WQS parameters and modeling strategies were specified as follows: First, regarding the validation strategy, a random 60:40 training-to-validation data split was applied. The training set (60% of the sample) was exclusively used to estimate the unknown component weights, while the holdout validation set (40%) was strictly reserved to test the statistical significance of the constructed WQS index, thereby effectively mitigating the risk of overfitting. Second, for the bootstrap settings, 200 bootstrap samples (with replacement) were generated within the training set to estimate robust empirical weights. Third, for quantile specification, the continuous hematological features were transformed into quartiles (*q* = 4) to limit the disproportionate influence of extreme outliers and place all predictors on a common standardized scale. Fourth, regarding directionality selection, because the true joint effect direction of the peripheral blood parameter cluster was *a priori* unknown, both positive (testing for a potential joint harmful effect) and negative (testing for a potential joint protective effect) directionalities were modeled independently. Model optimization was performed using the default non-linear programming solver (solnp algorithm), with convergence achieved when the fractional change in the objective function was less than the default tolerance threshold (10-5). Through this standardized procedure, a weighted index was constructed to quantitatively evaluate the overall joint effect of these indicators on the 30-day mortality risk in pediatric patients, adjusting for clinical covariates including age, sex, etiology, ICU admission, CNS involvement, PT, and APTT. Simultaneously, by extracting the mean empirical weights and their interquartile ranges (IQRs) generated from the bootstrap iterations, the relative independent contribution of individual hematological features to the prediction results was precisely quantified and the stability of the weights was verified. To explore the dose-response relationship between core continuous variables and the outcome, a restricted cubic spline (RCS) model was adopted to fit the potential non-linear association between the optimally selected, high-weight peripheral blood parameters and the 30-day mortality risk. Non-linear testing was utilized to assist in identifying potential clinical risk inflection points. Based on these core features, a predictive indicator for early mortality risk was constructed. Receiver operating characteristic (ROC) curves were plotted to evaluate the predictive efficacy of each single indicator and the combined model. The area under the curve (AUC) and its 95% confidence interval (CI) were calculated. The optimal clinical cut-off values were determined based on the principle of maximizing the Youden Index, with the corresponding sensitivity and specificity output simultaneously. To validate the clinical utility of the predictive indicators, decision curve analysis (DCA) was introduced. By calculating the clinical net benefit across different threshold probabilities, the actual benefit of each single indicator and the combined predictive indicator in guiding clinical decision-making was comparatively evaluated. Finally, to assess the robustness of the findings, an E-value sensitivity analysis was conducted for the key predictive indicators. The E-value refers to the minimum strength of association that an unmeasured confounder would need to have with both the treatment assignment and the outcome variable to explain away the observed point estimate or the lower limit of the 95% confidence interval (i.e., reducing the relative risk to 1.00) (13). Previous methodological studies suggest that an E-value >1.5–2.0 indicates a relatively strong resistance to unmeasured confounding. A two-sided *P*-value < 0.05 was considered statistically significant.

## 3 Results

### 3.1 Comparison of clinical characteristics between the survivor and non-survivor groups

A total of 133 pediatric patients with HLH were included in this study and divided into a Survivor group (*n* = 104) and a Non-survivor group (*n* = 29) based on their 30-day survival outcomes. Among the 29 non-survivors, all early mortality events were clinically considered HLH-related. The primary clinical drivers leading to death included multiple organ dysfunction syndrome (MODS) (*n* = 11), severe hemorrhage/disseminated intravascular coagulation (DIC) (*n* = 12), and refractory infections (*n* = 6).To account for potential confounding bias, patients were also classified according to HLH etiology. The cohort primarily consisted of infection-associated HLH (*n* = 81, 61%), followed by primary HLH (*n* = 28, 21%), malignancy-associated HLH (*n* = 16, 12%), autoimmune-associated HLH (*n* = 7, 5%), and metabolic disease-associated HLH (*n* = 1, 1%). The distribution of these etiological subtypes demonstrated no statistically significant difference between the Survivor and Non-survivor groups (*P* = 0.562). Furthermore, there were no significant differences in basic demographic characteristics, including sex and age, between the two groups (*P* > 0.05). However, patients in the non-survivor group exhibited higher rates of ICU admission (*P* < 0.001) and CNS involvement (*P* = 0.004), as well as significantly prolonged coagulation parameters including PT and APTT (both *P* < 0.001), indicating greater disease severity and coagulopathy. Regarding the peripheral blood laboratory findings, the level of RDW was significantly higher in the Non-survivor group (*P* < 0.05). Conversely, the levels of Hb, HCT, and MCH were significantly lower in the Non-survivor group (*P* < 0.05) (Table 1).

**Table 1:** Comparison of baseline characteristics and peripheral blood parameters between survivor and non-survivor groups in pediatric HLH.

Items	Total	Survivor Group	Non-survivor Group	Statistical Value	*P*
	(*n* = 133)	(*n* = 104)	(*n* = 29)		
Sex				0.333	0.564
Female	59 (44%)	48 (46%)	11 (38%)		
Male	74 (56%)	56 (54%)	18 (62%)		
Age	2.25 (1.00,5.00)	2.00 (1.00,4.87)	3.00 (1.00,6.00)	−0.501	0.616
Etiology, *n* (%)				1.957	0.745
Primary HLH	28 (21%)	22 (21%)	6 (21%)		
Infection-associated HLH	81 (61%)	65 (62%)	16 (55%)		
Malignancy-associated HLH	16 (12%)	11 (11%)	5 (17%)		
Autoimmune-associated HLH	7 (5%)	5 (5%)	2 (7%)		
Metabolicdisease-associated	1 (1%)	1 (1%)	0 (0%)		
ICU Admission, *n* (%)				22.690	<0.001
No	100 (75%)	88 (85%)	12 (41%)		
Yes	33 (25%)	16 (15%)	17 (59%)		
CNS Involvement, *n* (%)				8.281	0.004
No	118 (89%)	97 (93%)	21 (72%)		
Yes	15 (11%)	7 (7%)	8 (28%)		
WBC(×10^9^/L)	4.50 (2.51, 9.89)	4.45 (2.48, 8.26)	7.22 (2.90, 11.20)	−1.469	0.143
ANC(×10^9^/L)	1.33 (0.70, 3.30)	1.27 (0.70, 2.37)	2.45 (0.90, 6.27)	−1.806	0.071
LYM(×10^9^/L)	2.14 (0.93, 4.05)	1.95 (0.94, 3.46)	2.78 (0.80, 4.88)	−0.444	0.659
Monocytes(×10^9^/L)	0.43 (0.16, 0.85)	0.40 (0.16, 0.78)	0.50 (0.11, 1.47)	−0.886	0.377
RBC(×10^12^/L)	3.56 ± 0.83	3.59 ± 0.86	3.44 ± 0.70	1.030	0.327
Hb(g/L)	95.77 ± 18.48	97.66 ± 18.58	89.02 ± 16.69	2.095	0.036
HCT(%)	27.25 ± 5.65	27.87 ± 5.69	25.03 ± 4.99	2.624	0.009
MCH(pg)	26.00 (24.00, 27.30)	26.40 (24.50, 27.60)	24.50 (22.30, 26.00)	3.076	0.002
MCHC(g/L)	330.76 ± 16.49	329.61 ± 16.70	334.88 ± 15.26	−1.512	0.114
RDW(%)	15.20 (13.90, 18.90)	15.05 (13.70, 17.80)	16.80 (14.20, 19.80)	−2.201	0.028
Reticulocyte(%)	1.12 (0.50, 2.66)	1.12 (0.47, 2.72)	1.08 (0.80, 2.44)	−0.041	0.970
PLT(×10^9^/L)	62.00 (35.00, 100.00)	66.00 (37.00, 100.30)	41.30 (23.00, 92.70)	1.812	0.070
PT (s)	15.30 (13.10, 17.70)	14.9 (12.80, 16.70)	17.70 (15.40, 20.40)	−3.812	<0.001
APTT (s)	38.40 (30.70, 50.00)	36.10 (29.80, 44.90)	51.50 (41.00, 74.40)	−4.015	<0.001
Serum Ferritin (*μ*g/L)	1,808.00 (1,055.00,10195.00)	1,761 (996.00, 9,093.00)	7,290 (1,500.00,20923.00)	−1.832	0.067
Triglycerides (mmol/L)	3.15 (2.13, 4.74)	3.14 (2.12, 4.93)	3.19 (2.16, 4.04)	−0.793	0.428
NK cell activity (%)	5.59(3.49, 8.52)	5.38(3.28, 7.58)	7.11(4.03, 9.75)	−1.896	0.058

### 3.2 Univariate preliminary screening and robustness assessment of key prognostic parameters

The preliminary screening results from the univariate logistic regression analysis revealed that an elevated RDW was significantly associated with an increased risk of early mortality in pediatric HLH (OR = 1.118, 95% *CI*: 1.002–1.248, *P* = 0.046). MCH at admission was significantly associated with a lower short-term mortality risk (*OR* = 0.856, 95% *CI*: 0.759–0.965, *P* = 0.011); additionally, elevated levels of HCT (*OR* = 0.913, 95% *CI*: 0.847–0.985, *P* = 0.019) and Hb (*OR* = 0.974, 95% *CI*: 0.952–0.997, *P* = 0.029) were both closely associated with better early survival outcomes.To evaluate the robustness of the aforementioned univariate associations against potential unmeasured clinical confounders, the E-value was calculated for each significant variable. The results demonstrated that the E-values for MCH and RDW reached 1.612 and 1.482, respectively, while the E-values for HCT and Hb reached 1.416 and 1.191, respectively (Table 2).

**Table 2:** Univariate logistic regression and sensitivity analysis of red blood cell parameters for predicting 30-day mortality in pediatric HLH.

Items	*β*	*SE*	Wald*χ*2	*P*	*OR*(95%*CI*)	E-value for *OR*	E-value for lower
HCT	−0.090	0.039	5.512	0.019	0.913 (0.847–0.985)	1.416	1.139
MCH	−0.156	0.061	6.423	0.011	0.856 (0.759–0.965)	1.612	1.229
RDW	0.112	0.056	3.990	0.046	1.118 (1.002–1.248)	1.482	1.048
Hb	−0.026	0.012	4.773	0.029	0.974 (0.952–0.997)	1.191	1.055

### 3.3 Joint effect and weight analysis of peripheral blood parameters based on WQS regression

**Figure 2:**
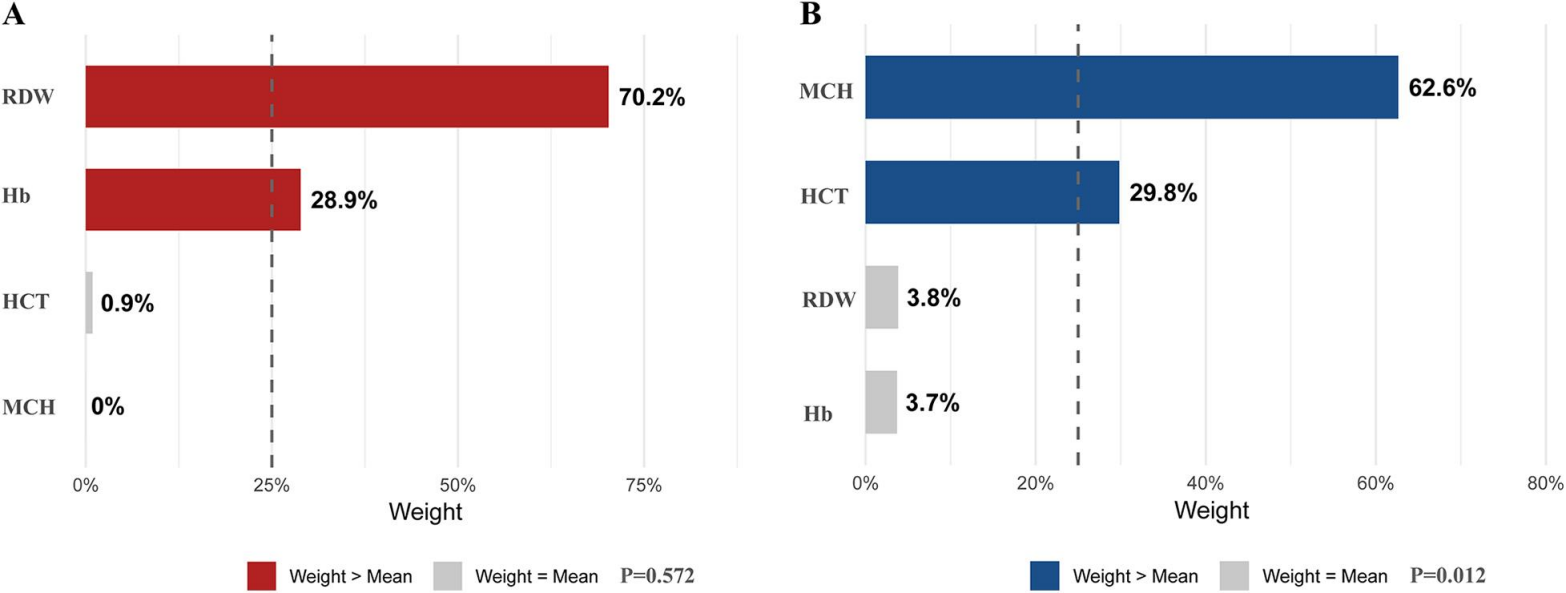
Relative empirical weights of peripheral blood parameters in the multivariable adjusted WQS models for predicting 30-day mortality. **(A)** Weight distribution in the positive-direction WQS model evaluating potential risk effects. **(B)** Weight distribution in the negative-direction WQS model evaluating potential protective effects. Each bar represents the empirical weight of an individual parameter estimated from the training dataset partition (60% of the total sample), indicating its relative contribution to the overall composite index. The vertical dashed line represents the mean weight threshold (0.25). Parameters with weights exceeding this threshold are considered primary contributors to the joint effect. To address the uncertainty inherent in the finite sample size, the stability of these weights was strictly verified using the IQR derived from 200 bootstrap iterations, confirming MCH (62.6%) and HCT (29.8%) as the statistically robust primary drivers. Both models were adjusted for relevant clinical covariates including ICU admission, CNS involvement, PT, and APTT. WQS, weighted quantile sum; MCH, mean corpuscular hemoglobin; HCT, hematocrit; RDW, red blood cell distribution width; Hb, hemoglobin.

To mitigate collinearity interference among peripheral blood parameters and to adjust for potential clinical confounders related to disease severity, a WQS regression model was constructed based on a 60:40 training-to-validation split to ensure robust estimation. Crucially, this multivariable WQS model incorporated ICU admission, CNS involvement, PT, and APTT as clinical covariates. The results demonstrated that in the validation dataset, the positive-direction WQS model assessing potential risk effects showed no statistically significant association between the constructed WQS index and mortality risk (*β* = 0.206, *P* = 0.572) (Figure 2A). Conversely, when evaluated in the validation dataset, the negative-direction WQS model revealed that the WQS index was significantly and negatively associated with the 30-day mortality risk in pediatric patients (*β* = −0.936, *P* = 0.012), indicating that the overall improvement of red blood cell parameters exerts a significant joint protective effect on short-term prognosis independent of severe clinical conditions (e.g., ICU admission, CNS involvement, and coagulopathy). By extracting the empirical weights estimated from the training dataset via 200 bootstrap iterations and calculating their IQRs to assess stability, it was revealed that MCH (weight: 62.6%, IQR: 47.6%–75.3%) and HCT (weight: 29.8%, IQR:12.8%–46.2%) at admission exhibited the highest relative weights within the evaluated mixture (Figure 2B).

### 3.4 Dose-response relationship analysis of peripheral blood parameters based on RCS

**Figure 3:**
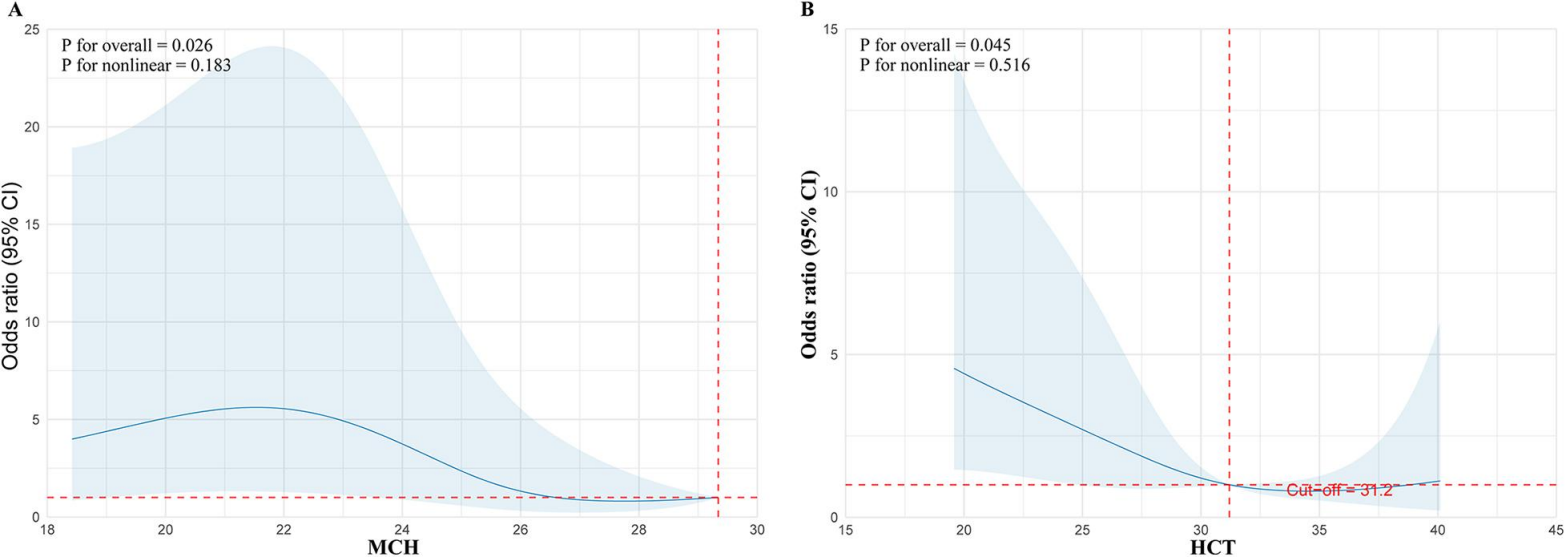
Dose-response relationships between core peripheral blood parameters and 30-day mortality risk in pediatric HLH based on RCS. **(A)** Association between MCH at admission and mortality risk. **(B)** Association between HCT at admission and mortality risk. Solid lines represent the estimated OR for mortality, and the shaded areas denote the 95% CI. A non-linear *P*-value > 0.05 indicates an approximately linear dose-response relationship. The vertical dashed lines delineate the identified optimal clinical cut-off values. HLH, hemophagocytic lymphohistiocytosis; RCS, restricted cubic spline; OR, odds ratio; CI, confidence interval; MCH, mean corpuscular hemoglobin; HCT, hematocrit.

To explore the quantitative relationship between the core peripheral blood parameters optimally selected by the WQS regression model and the 30-day mortality risk in pediatric HLH, a RCS model was constructed to fit their potential dose-response curves. The results demonstrated a significant overall statistical association between MCH at admission and the short-term mortality risk of the patients (*P*_over−all_ = 0.026). The non-linear test indicated an approximately linear relationship (*P*_non−linear_ = 0.183) (Figure 3A). Similarly, a significant overall statistical association was observed between HCT at admission and the short-term mortality risk (*P*_over−all_ = 0.045), with the non-linear test also suggesting an approximately linear relationship (*P*_non−linear_ = 0.516) (Figure 3B).

### 3.5 Clinical utility assessment based on DCA

**Figure 4:**
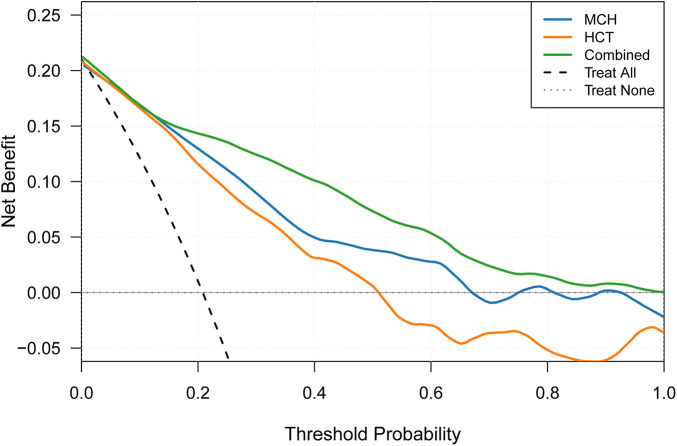
DCA evaluating the clinical utility of peripheral blood parameters for predicting 30-day mortality in pediatric HLH. The *x*-axis represents the threshold probability, and the *y*-axis represents the net clinical benefit. The combined predictive indicator incorporating MCH and HCT (Combined) consistently demonstrates a higher net benefit across a broad range of threshold probabilities compared to the single parameter models (MCH or HCT alone) and the extreme clinical baseline assumptions of treating all patients (Treat All) or treating no patients (Treat None). HLH, hemophagocytic lymphohistiocytosis; DCA, decision curve analysis; MCH, mean corpuscular hemoglobin; HCT, hematocrit.

To validate the clinical decision-making value of the core red blood cell parameters identified through the WQS regression model in a real-world setting, this study employed DCA to quantitatively evaluate the clinical net benefit of each indicator and the combined model. The results demonstrated that within the threshold probability range of 0.1–0.8 (reflecting the clinical risk tolerance for missing a potentially fatal early outcome vs. the cost of triggering unnecessary aggressive interventions), the clinical net benefit curve of the combined predictive indicator (comprising MCH and HCT) consistently remained at the top. The net benefit of this combined model was not only significantly higher than the extreme clinical baseline assumptions of “Treat All” or “Treat None” but also markedly superior to predictive indicators based on single hematological parameters, such as MCH or HCT alone (Figure 4).

### 3.6 Construction and efficacy evaluation of early mortality risk predictive indicators based on peripheral blood parameters

**Figure 5:**
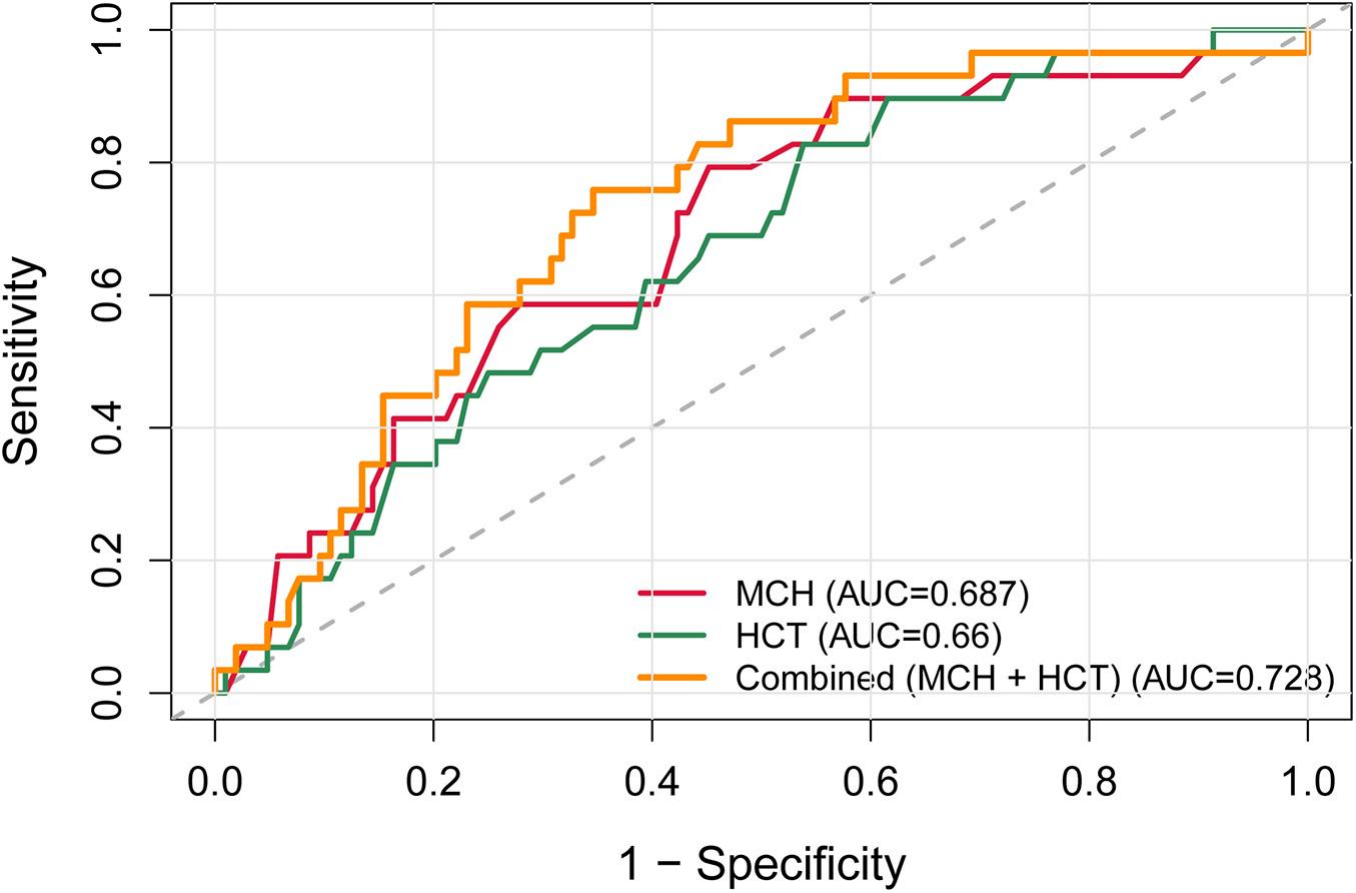
ROC curves of individual peripheral blood parameters and their combined model for predicting 30-day mortality in pediatric HLH. The predictive efficacy of MCH, HCT, and their combined model (Combined: MCH + HCT) were evaluated. The combined model exhibited the highest AUC (0.728), indicating superior discrimination capacity for early mortality prediction compared to any single indicator. HLH, hemophagocytic lymphohistiocytosis; ROC, receiver operating characteristic; AUC, area under the curve; MCH, mean corpuscular hemoglobin; HCT, hematocrit.

To evaluate the application value of MCH and HCT in predicting the 30-day mortality risk in pediatric HLH, this study quantitatively assessed their predictive efficacy using ROC curves. The results demonstrated that the AUC for the single indicators MCH and HCT in predicting early mortality in pediatric patients were 0.687 (95%*CI*: 0.579–0.795) and 0.660 (95%*CI*: 0.554–0.765), respectively. To optimize the diagnostic performance of the model, this study combined MCH and HCT to construct a composite risk predictive indicator. The results revealed that the comprehensive predictive efficacy of the combined model was significantly enhanced, with its AUC increasing to 0.728 (95%*CI*: 0.629–0.827) (Table 3 and Figure 5).

**Table 3:** Predictive performance of peripheral blood indicators and their combined model for 30-day mortality in pediatric HLH.

Items	AUC (95% *CI*)	Cutoff	Sensitivity	Specificity	Accuracy	PPV	NPV
MCH	0.687 (0.579–0.795)	26.050	0.793	0.548	0.602	0.329	0.905
HCT	0.660 (0.554–0.765)	29.350	0.828	0.462	0.541	0.300	0.906
Combined (MCH + HCT)	0.728 (0.629–0.827)	0.208	0.759	0.654	0.677	0.379	0.907

Combined model includes MCH and HCT.

## 4 Discussion

Although the popularization of salvage therapies, such as hematopoietic stem cell transplantation, has significantly improved the long-term survival expectations of children with HLH, it has not completely resolved the clinical dilemma of high early (30-day) mortality following diagnosis. During this critical intervention window, clinicians lack evaluation tools that effectively integrate multidimensional clinical information while overcoming the multicollinearity of biological data. Consequently, some pediatric patients face rapid disease deterioration or multiple organ failure before induction therapy takes effect. Our analysis underscores that within the complex physiological stress of pediatric HLH, hematological parameters function as a synchronized network rather than isolated variables, where their inherent multicollinearity often renders traditional single-biomarker or linear evaluation systems inadequate. By employing the WQS regression model, we were able to effectively account for these joint effect trajectories and circumvent the statistical bias of traditional models when assessing 30-day mortality risk. The results demonstrate that abnormalities within the red blood cell parameter cluster—predominantly weighted by HCT and MCH—act as an exploratory composite indicator for identifying patients at high risk of early fatal outcomes.

This study revealed that HCT exhibited a prominent relative weight within the joint mixture evaluated by the WQS model. Because our retrospective clinical dataset did not directly include underlying molecular biological markers (such as specific inflammatory cytokines), we propose plausible pathophysiological hypotheses for the mechanisms underlying these data associations based on previous literature. The core immunodeficiency of HLH lies in the severe functional impairment of cytotoxic T lymphocytes and natural killer cells, leading to the failure of antigen clearance and triggering the continuous and excessive activation of macrophages (14). During this pathological process, a massive release of a pro-inflammatory cytokine storm, primarily driven by high concentrations of interferon-gamma (IFN-*γ*), theoretically not only directly drives the polarization and hyper-phagocytic activity of tissue macrophages but also prompts them to extensively and non-specifically phagocytose normal autologous red blood cells within the reticuloendothelial system, such as the bone marrow and spleen, further inducing severe erythroid hematopoietic failure (15). Concurrently, it is well-documented in existing literature that high levels of tumor necrosis factor-alpha (TNF-α) and interleukin-6 (IL-6) strongly inhibit compensatory bone marrow erythropoiesis by inducing apoptosis in hematopoietic stem/progenitor cells and interfering with the receptor signal transduction of erythropoietin (EPO) (16). We hypothesize that this dual pathological mechanism—accelerated peripheral red blood cell destruction combined with central hematopoietic suppression—leads to a progressive decrease in HCT during the acute phase of the disease.

Similarly, MCH also demonstrated a prominent relative weight within the evaluated mixture. Unlike anemia caused by acute blood loss, the characteristic microcytic hypochromic anemia observed in pediatric HLH is postulated to be attributed to inflammation-induced iron metabolism reprogramming mechanisms. Although iron profiles and hepcidin levels were not directly measured in our cohort, established pathophysiological models suggest that a persistently high inflammatory state stimulates the liver to upregulate hepcidin expression, which subsequently blocks iron release from the reticuloendothelial system by degrading ferroportin, resulting in functional iron restriction within the erythroid hematopoietic system despite adequate iron stores (17). Because alterations in red blood cell morphology require a relatively long metabolic cycle, the reduction in MCH suggests that the patients had likely already endured continuous immune attacks and depletion of physiological reserves during the insidious phase prior to diagnosis. This state of chronic physiological exhaustion indicated by reduced MCH intertwines with extensive organ infiltration marked by hepatomegaly and the acute inflammatory burden indicated by a surge in C-reactive protein (CRP), jointly constituting a multiplicative effect on mortality risk (18). As a key methodological highlight of this study, the statistical robustness of these core hematological findings is strongly supported by our E-value sensitivity analysis. Taking MCH as an example, an unmeasured confounder would need to be associated with both MCH levels and the early mortality risk by a risk ratio of at least 1.612 to fully explain away the observed protective associations. This quantitative evidence demonstrates that our primary findings maintain a high degree of resistance to unmeasured confounding biases, reinforcing their reliability as robust clinical warning signals in the acute phase of pediatric HLH.

In summary, although the WQS regression model constructed in this study demonstrates robust discriminatory power and clinical interpretability in revealing the joint effects of peripheral blood indicators, several limitations remain. First, regarding the study design, the inherent nature of a single-center retrospective cohort inevitably introduces selection and information biases. Restricted by historical electronic medical record data, potential unmeasured confounding factors were difficult to control completely, and the regional demographic characteristics of a single center may limit the direct generalizability of these findings to populations with different ethnicities or levels of medical resources. Second, despite employing a rigorous 60:40 training-to-validation split and 200 bootstrap iterations to mitigate overfitting and support the stability of the component weights, the relatively small total sample size (*n* = 133) and limited number of early mortality events (*n* = 29) inherently constrain the overall statistical power. Formal clinical prediction models must balance the number of candidate predictors against expected outcome events to minimize optimism and enhance calibration precision. Consequently, the restricted event count in our cohort inherently increases the risk of overfitting. It should also be acknowledged that formal internal calibration, such as bootstrap optimism correction—a standard step in prediction model development—was not performed in this study. Due to this restricted event count, we utilized univariate pre-screening prior to WQS modeling to facilitate algorithmic convergence. We acknowledge that this approach may introduce selection bias by inadvertently excluding variables that lack individual marginal effects but possess potentially important synergistic prognostic value. Consequently, the AUC and DCA findings of our predictive model must be interpreted with strict caution. The current findings serve primarily as exploratory, proof-of-concept evidence, underscoring the urgent need for large-scale, prospective external validation to confirm these joint trajectories. Third, and perhaps most critically regarding potential confounders, our models were unable to achieve true, comprehensive multivariable clinical adjustment. We successfully incorporated several important covariates (HLH etiology, ICU admission, CNS involvement, PT, and APTT) to partially account for disease severity and coagulopathy. Still, other established biological determinants were not included in the final WQS prognostic model. Specifically, although core HLH disease activity markers such as serum ferritin and triglycerides were assessed at baseline, they did not reach statistical significance during the preliminary univariate screening—a limitation likely attributable to the constrained sample size and restricted event count. Furthermore, due to the retrospective design, other critical variables, including fibrinogen, soluble CD25, detailed treatment timing (e.g., treatment delay and specific HLH-directed induction therapies), precise infection severity scores, and critical fluid-status events (such as recent blood transfusion status), could not be systematically collected and adjusted for. Without comprehensively modeling these paramount confounders, we cannot definitively assert that the observed alterations in HCT and MCH represent entirely independent prognostic biology. Instead, as the reviewers insightfully noted, these lower red blood cell parameters may partially reflect greater overall disease severity, severe DIC/bleeding cascades, inflammatory suppression of erythropoiesis, active hemophagocytosis, or transfusion-related hemodilution. Fourth, pediatric HLH possesses profound etiological heterogeneity. For certain rare etiologies (such as metabolic diseases or specific autoimmune-triggered HLH), the current model cannot perfectly capture their unique prognostic trajectories due to limited sample sizes, making it more of a broad-spectrum early warning tool rather than an etiology-specific prognostic standard.

Despite these limitations, the indicators selected in this study offer potential additive clinical value, particularly for frontline pediatricians facing acute onset. We acknowledge that the combined model exhibits only modest discrimination (AUC=0.728); therefore, it is not intended to replace comprehensive clinical judgment but rather to serve as an adjunctive alert.The target population for this exploratory analysis is precisely defined as pediatric patients with newly diagnosed HLH who require urgent risk stratification before the initiation of specific induction therapy. By relying on easily accessible routine biomarkers, this study provides a hypothesis-generating signal that can assist in identifying children facing an extremely high risk of death within 30 days. Importantly, our DCA results provide essential clinical context for this signal. The combined model demonstrates a positive net benefit across a broad range of threshold probabilities (10% to 80%). In the critical clinical context of pediatric HLH, the cost of a false negative (missing a patient at high risk of early death) is catastrophic, whereas the cost of a false positive (closer monitoring or pre-emptive ICU transfer) is relatively acceptable. Therefore, while the mathematical optimal cut-off based on the Youden index was 0.208, we recommend adjusting the practical clinical decision threshold to a lower risk tolerance range (e.g., 0.15). A threshold of 0.15 is clinically justified because it implies that if a patient's predicted risk of early mortality reaches 15%, the survival benefit of aggressive early intervention fundamentally outweighs the resource burden and potential harms of over-triage. In real-world clinical triage, this threshold serves as an actionable early warning trigger: when a newly diagnosed pediatric patient's composite index meets or exceeds this 15% mark, frontline clinicians should avoid a wait-and-see approach. Instead, they should immediately escalate the level of care by initiating continuous vital sign monitoring, proactively consulting the pediatric intensive care unit for pre-emptive transfer, and preparing for the urgent initiation of HLH-directed therapies. This safety-first approach inherently prioritizes sensitivity, facilitating the early capture of a broader range of potentially high-risk pediatric patients for timely transfer to intensive care and the escalation of individualized immunomodulatory therapy. However, before this combined model can be considered clinically actionable, further rigorous internal validation, external multicenter validation, and, crucially, comprehensive adjustment for established HLH severity markers (e.g., ferritin, fibrinogen, triglycerides, sCD25) alongside detailed transfusion and treatment delay records, are strictly required. Looking ahead, future research should focus on conducting large-scale, multicenter prospective cohort studies to rigorously test the external robustness of the model. Additionally, expanding single baseline data to include dynamic monitoring of key variables throughout the disease course, and integrating multi-omics data such as genomics, transcriptomics, and proteomics, will be essential steps in upgrading this tool into a dynamic, multi-dimensional precision medicine evaluation system. It is important to acknowledge that the detailed biological mechanisms discussed herein are extrapolated from existing literature rather than directly derived from the current dataset. Finally, it should be noted that the empirical weights derived from the WQS model represent only the relative statistical contribution of each indicator within this specific evaluated mixture, rather than their absolute causal importance. Because the model remains essentially a statistical evaluation tool based on observational data associations, basic experimental studies are strictly required to verify the direct causal biological pathways behind these associations.

## 5 Conclusion

The analytical strategy incorporating the WQS regression model effectively mitigates multicollinearity interference among clinical variables. Abnormalities in peripheral blood parameters, primarily driven by HCT and MCH, serve as exploratory composite indicators for predicting early fatal outcomes in pediatric HLH. This composite predictive model can capture early signals of cumulative physiological exhaustion, providing predictive efficacy and clinical net benefit superior to single indicators. Importantly, we explicitly emphasize that this predictive model is strictly exploratory and is not yet ready for clinical application. While the findings are hypothesis-generating, rigorous external multicenter validation and comprehensive adjustment for established HLH severity markers are required before this tool can be used in clinical practice.

## Data Availability

The raw data supporting the conclusions of this article will be made available by the authors, without undue reservation.
